# The annual, temporal and spatial pattern of *Setaria tundra* outbreaks in Finnish reindeer: a mechanistic transmission model approach

**DOI:** 10.1186/s13071-018-3159-z

**Published:** 2018-11-12

**Authors:** Najmul Haider, Sauli Laaksonen, Lene Jung Kjær, Antti Oksanen, Rene Bødker

**Affiliations:** 1 National Veterinary Institute, Technical University of Denmark, Kgs. Lyngby, Denmark; 2 Department of Veterinary Biosciences, Faculty of Veterinary Medicine, University of Helsinki, Helsinki, Finland; 3 WAZAMA Media Oy, Kuusamo, Finland; 4 Finnish Food Safety Authority Evira (FINPAR), Oulu, Finland

**Keywords:** *Setaria tundra*, Spatio-temporal distribution, Disease outbreak, Reindeer, Finland, Microclimatic temperature, Climate change

## Abstract

**Background:**

In northern Finland (Lapland), reindeer are reared as semi-domesticated animals. The region has a short summer season of 2–3 months, yet reindeer are infected with the mosquito-borne filarioid parasite *Setaria tundra*. The infection causes peritonitis and perihepatitis, which cause significant economic losses due to reduced body weight of infected animals. The objective of this study was to: (i) describe the spatial and temporal pattern of outbreaks in three different areas across Finnish Lapland; and (ii) construct a temperature-driven mechanistic transmission model to quantify the potential role of temperature on intensity of *S. tundra* transmission in reindeer.

**Methods:**

We developed a temperature-driven transmission model able to predict the number of *S. tundra* potentially transmitted from an infectious reindeer. We applied the model to the years 2004–2015, and compared the predictions to the proportion of reindeer whose livers were condemned due to *S. tundra* infection at the time of slaughter.

**Results:**

The mean proportion of liver condemnation increased in reindeer slaughtered in late autumn/winter compared to earlier dates. The outbreaks were geographically clustered each year but there were no fixed foci where outbreaks occurred. Larger outbreaks were recorded in the southern regions of reindeer-herding areas compared to the central or northern parts of Lapland. Our model showed that temperatures never allowed for transmission of more than a single generation of *S. tundra* each season. In southern (Kuusamo) and central (Sodankylä) Lapland, our model predicted an increasing trend from 1979 to 2015 for both the duration of the effective transmission period of *S. tundra* (*P* < 0.001) and for the potential number of L3 *S. tundra* larvae being transmitted from an infectious reindeer (*P* < 0.001).

**Conclusions:**

The effective transmission period for *S. tundra* in reindeer is very short in Lapland, but it increased over the period studied. Only one generation of *S. tundra* can be transmitted in one season among reindeer in Lapland. Increasing temperatures may facilitate a range expansion and increasing duration of effective transmission period for *S. tundra*.

**Electronic supplementary material:**

The online version of this article (10.1186/s13071-018-3159-z) contains supplementary material, which is available to authorized users.

## Background

An increase in outbreaks of mosquito-borne filarial *Setaria tundra* infection has been documented in Finnish reindeer (*Rangifer tarandus tarandus*) husbandry in recent years [1]. The disease is associated with peritonitis, perihepatitis and poor body condition [1–4]. A correlation has been found between the adult worm load in the abdominal cavity and the degree of peritonitis/perihepatitis in slaughtered reindeer [5]. At least three large outbreaks of *S. tundra* in ungulates have been documented, in 1973, 1989 and 2003 [1]. These outbreaks were all associated with relatively warm summers, and a relationship between climate change and increasing *S. tundra* outbreaks in Finnish reindeer has been suggested [3]. Studies also showed a correlation between higher mean temperatures of two successive summers and *S. tundra* outbreaks in Finland [3]. Many other studies on vector-borne diseases have suggested a similar correlation between increasing temperatures and disease outbreaks [6–9]. The exact mechanism is not well described, but may include: (i) an increase in the duration of the annual transmission periods, allowing more generations of the pathogens; (ii) shortened pathogen development time in vectors; and (iii) and increased vector abundance

After ingesting microfilariae from an infected reindeer, the mosquitoes must survive long enough for the microfilariae to develop into infective larval stage 3 (L3). The development time from microfilaria to L3, known as the extrinsic incubation period (EIP), depends on the environmental temperature [10, 11]. There are no available data on the relationship between temperature and development rates of *S. tundra* L3 in mosquitoes, though studies on the mosquito-borne filarial worms *Dirofilaria immitis* and *D. repens* have generally found 14 °C to be the lower threshold temperature for development into L3 stages [12–14]. Laaksonen et al. [2] found that microfilaria develops to the infective L3 stage in mosquitoes over approximately 14 days at a mean temperature of 21 °C, whereas development was not completed at a mean temperature of 14.1 °C. After completion of the EIP, mosquitoes transmit the L3 to a new susceptible host, where it develops into an adult worm in approximately 2–4 months [2]. The adult male and female worms mate to generate the microfilaria offspring [2]. It is thought that the microclimatic temperature at the vector resting sites has an important impact on the transmission of vector-borne diseases in northern Europe [10, 15]. The reindeer-herding regions of Finnish Lapland have cool and very short summers (around 2–3 months), but regular, small *S. tundra* outbreaks have recurred here since 2003 [1–4].

There are 54 reindeer cooperatives in Finland [16]. Reindeer from different cooperatives often mix while grazing in adjoining areas. There are usually several herds (~ 2–4) of animals in the same cooperative, and herd owners allow them to mix with reindeer from other owners within the cooperative. Animals selected for breeding are treated with the antiparasitic drug Ivermectin in autumn and released. Most of the reindeer are slaughtered from September to December, but a small proportion may be slaughtered as late as the following April. Of the slaughtered reindeer, 80% were born the same year and 20% are old, usually around 8–9 years-old [5]. Most of the reindeer (~ 70–80%) are slaughtered in approved abattoirs and the rest are slaughtered privately [5, 17]. Meat inspection has occurred regularly since the 1980s [5, 17], while data on liver condemnation due to *S. tundra* and other causes have only been recorded precisely since 2004.

*Aedes* spp. and to some extent *Anopheles* spp. mosquitoes are the main vectors of *S. tundra* [2]. Unfortunately, the abundance of these mosquito species is not well documented in Finland. Some estimates suggest that reindeer can be exposed to attacks by approximately 8000 mosquitoes an hour during some periods [18]. In the Kuusamo region, as many as 426 mosquitoes were caught in hand nets per min during the first week of August [1]. *Setaria tundra* microfilaremia also varies throughout the year in reindeer, with the peak period from mid-June to late August [4], with the peak abundance of microfilaremia in reindeer and peak mosquito activity in Finland coinciding [2].

The objective of this study was to: (i) describe the spatial and temporal pattern of outbreaks at three different latitudes in northern Finland; and (ii) construct a temperature-driven mechanistic transmission model to quantify the potential role of temperature on the intensity of *S. tundra* transmission.

## Methods

### Grouping of cooperatives

We grouped cooperatives close to three different weather stations in Kevo (north), Sodankylä (central) and Kuusamo (south) in order to calculate the mean proportion of organ condemnation and to estimate the worm transmission with a model based on meteorological data from each of the three stations . The northern region included cooperatives 1, 2, 3, 4, 5, 6, 7, 8 and 10, the central region included cooperatives 15, 16, 17, 18, 19, 21 and 22, and the southern region included cooperatives 24, 25, 26, 35, 36, 37, 38, 42, 43, 45, 46, 48 and 51.

### Liver condemnation due to *S. tundra* infection in reindeer

We collected the reindeer meat inspection data from the slaughterhouses for the period 2004–2015. Meat inspection and hygiene control are conducted at slaughterhouses by the regional state administrative agencies of Finnish Lapland [17]. The meat inspection procedure has previously been described in detail [5, 17]. In short, the degree of liver damage caused by *S. tundra* is measured on a scale of 0–3. There is a correlation between the number of adult worms living in the abdominal cavity of the reindeer and the condition of their livers [5]. We considered organ changes with local fibrin formation on the surface of the liver or peritoneum resulting in condemnation the whole organ (liver) (scale 2–3) as *S. tundra* infection [5]. We calculated the prevalence of *S. tundra* in reindeer as the proportion of animals whose whole livers were condemned due to *S. tundra* compared to the total number of reindeer inspected at the slaughterhouse.

### Meteorological temperature data

We collected data on the daily maximum, minimum and mean temperatures from the Finnish Meteorological Institute (FMI) for the period 1979–2015. Data were recorded at three weather stations located in three different regions. We converted the daily maximum and minimum temperatures into hourly temperatures using a simple sinusoidal distribution as described by Parton & Logan [19].

### Microclimatic temperature data

Microclimatic temperature is considered to be useful for estimating vector-borne disease transmission [10, 15, 20], but these data were not available from the study areas. A recent study of the Danish climate concluded that microclimatic habitats were on average 3.5–5.0 °C warmer at midday than the meteorological recorded temperatures, but 1–3 °C cooler at midnight [10]. In accordance with this finding, we estimated the microclimatic temperature by adding 3.5 °C to the daily maximum temperature and deducting 1 °C from the daily minimum temperature, then converted them to hourly temperatures using a sinusoidal distribution [19].

### Mosquito abundance data

Few data on vector abundance are available for northern Finland. Laaksonen et al. [1] collected mosquitoes using a hand net for 60 s every week during summer 2004 in the Kuusamo region of Finland and identified three genera (*Aedes*, *Anopheles* and *Culiseta*). In the present study, we used the data from Laaksonen et al. [1] (sum of *Aedes* spp. and *Anopheles* spp.) to estimate mosquito abundance. We used a running average of 30 days to smooth the distribution (Additional file 1: Figure S1). With no other data available, we assumed the smoothed vector abundance recorded in 2004 would be representative of all years in order to capture the main seasonal trend. Any variation in vector abundance from year to year was thus ignored in the analysis of transmission (Additional file 1: Figure S1).

### Microfilarial density data

We used published *S. tundra* microfilaria data monitored at Oulu Zoo in 2004 [4]. In March 2004, three male and four female reindeer were relocated from Kuusamo to the Oulu Zoo area and were naturally infected with *S. tundra.* The microfilariae were monitored weekly for one year by collecting blood from a jugular vein, as described by Laaksonen et al. [4]. To obtain a daily observation, we calculated a 30-day running average of the microfilaria data, again assuming that the microfilaria data recorded in 2004 were representative of all years. Any variation in microfilarial density from year to year was therefore ignored in the analysis of transmission (Additional file 1: Figure S1).

### Mechanistic model for estimating the number of worms transmitted from an infectious reindeer

The following terminology is used:

- *Extrinsic Incubation Period (EIP)*: the temperature-dependent time interval between a mosquito receiving blood meals infected with *S. tundra* microfilariae to the microfilariae developing into infective L3 larvae.
- *Duration of the gonotrophic cycle*: the interval between two successive blood meals taken by a mosquito.
- *Daily survival probability*: here the survival of mosquitoes is assumed to depend only on daily mean temperature. We used a mathematical equation in which the daily survival rate of mosquitoes was dependent on temperature [21], and restricted the daily survival rate to a maximum of 90%. We considered the maximum lifespan of mosquitoes to be 60 days.
- *Effective period for S. tundra transmission from host to vector*: the number of days in a year when mosquitoes receive an infected blood meal (with microfilariae of *S. tundra*) and survive long enough for the ingested worms to develop into L3 larvae that can be transmitted to a new host.

We developed a mechanistic model to estimate the potential number of *S. tundra* L3 transmitted *via* vectors from one infectious reindeer to other reindeers during one season. In this manuscript, we call this the potential number of L3 transmitted. We assumed that the infectious reindeer would be present throughout the year and that the reindeer were bitten daily by a number of mosquitoes estimated from the smoothed observed data from 2004.

The model is designed to follow cohorts of biting mosquitoes each day throughout the season at the temperatures recorded by the FMI weather stations in the three different regions (south, central and north).

In the model, mosquitoes take a blood meal infected with *S. tundra* microfilariae, rest until the gonotrophic cycle is completed and then successfully take a new blood meal. At each bite, mosquitoes consume 0.005 ml blood [22] with the daily density of microfilariae recorded in 2003–2004 [4]. Completion of the EIP is solely dependent on the hourly temperature experienced each day by the cohort of mosquitoes. After the EIP is completed, we assumed that the mosquitoes would transmit all the L3 larvae during the first bite, and that subsequent bites would therefore not be infectious. We developed a rate summation model in which the EIP or blood meal digestion rate is calculated hourly and summed up daily until the parasite development/blood meal digestion is complete. The three temperature-dependent equations used in the model are listed in Table 1.

**Table 1:** The equations used to model changes in the extrinsic incubation period (EIP) of *S. tundra* in mosquitoes, blood-meal digestion period (biting rate) and daily survival rate of mosquitoes

Trait	Equation	Reference
EIP of *S. tundra* in mosquitoes	1/((T-14)/130)	[10, 12, 13]
Blood meal digestion rate (biting rate)	1/(0.0943 + 0.0043*T)	[32]
Survival rate of mosquitoes	e^-1/(-4.4 + 1.31*Tmean – 0.03*(Tmean)^2)^	[21, 33]
	Daily maximum survival rate is set as 90%	

*Abbreviations*: T, temperature (hourly, °C); Tmean, daily mean temperature (°C)

The steps in the model are described below:

1. The model follows a daily cohort of vectors. A cohort is defined here as the number of vectors biting an infectious reindeer host on a given day. The model follows this cohort until all vectors are dead, with the maximum vector survival set to 60 days. The model runs for one cohort at a time, starting with the cohort biting the first day of the selected time period and moving successively through the days of the remaining time period. During each run, the model calculates three different events (daily survival rate, EIP and biting rate) in the life of each cohort.
2. Based on the successive average temperature for each daily cohort, the model calculates the number of mosquitoes surviving to the following day for a maximum period of 60 days (Table 1).
3. The model calculates the EIP of *S. tundra* (Table 1) based on the successive hourly temperatures for each daily cohort, and identifies the date when the mosquitoes in each cohort become infectious, i.e. when the EIP is completed.
4. The model calculates and identifies the dates when the vectors complete each gonotrophic cycle (Table 1). It is assumed that the mosquitoes will take a new blood meal on the same day that each gonotrophic cycle is completed.
5. The model identifies the dates of the infectious bites and then merged them with the information on survival rates to calculate how many vectors of the original cohort have survived until that day. The number of surviving vectors represents the number of new infectious bites by the vectors in the specific cohort.
6. The model gives three estimates: (i) the date the cohort became infected; (ii) the date that bites from the cohort result in infection; and (iii) the number of infectious bites produced by the cohort each day. Based on the microfilarial density in reindeer on the date the vector became infected, the model can estimate how many L3 larvae are transferred on the date the vectors give the infectious bites.
7. When all the daily cohorts are processed in the model, the number of infectious L3 transmitted from each cohort are summarized by date, giving the total number of L3 transmitted per day throughout the season and originating from one infectious reindeer. For example, a cohort of mosquitoes may complete eight gonotrophic cycles in their lifetime, but if it is too cold for the EIP to complete within the lifetime of the cohort, the mosquitoes will not contribute to *S. tundra* transmission. However, if the EIP is completed between the fifth and sixth gonotrophic cycles, the surviving mosquitoes of that cohort will transmit L3 larvae when they take their sixth blood meal. In this case, the model further assumes that there would be no worms left to transmit during the seventh and eigth blood meals. This differs from other vector-borne infectious disease models (e.g. the basic reproduction rate model for bluetongue and Schmallenberg virus [23, 24]), in which all bites are infectious after the EIP is completed, but the virus load is not quantified.
8. We considered that each worm would contribute to detectable damage and potential condemnation at the slaughterhouses 75 days after infecting a reindeer based on observed onset of ogran condemnation on slaughterhouses

### Data analysis

We estimated the weekly and monthly mean proportion of liver condemnation due to *S. tundra* using slaughterhouse records for all cooperatives in the three regions (southern, central and northern parts of Lapland). For each cooperative, we calculated the mean annual proportion of liver condemnation and the average condemnation over 12 years (2004–2015). The estimates for each cooperative were plotted on a map in QGIS [25]. We plotted the maximum weekly proportion of liver condemnation at each region against the average cumulative daily number of L3 estimated by the model to be transmitted from an infectious reindeer for each of the three regions.

We selected maximum weekly liver condemnation because our model is based on the assumption that infectious reindeer are present at the beginning of and throughout each season. But the model does not take into account how many infectious reindeer are present as such data were not available. Proportion of liver condemnation will only be high when both the transmission potential is high and the number of infectious reindeer is high. Herds with no or a small proportion of infectious reindeer during the season would end up with no or very low condemnation rates, even if the model predicts a high transmission potential. By selecting the maximum weekly condemnation rate we insured that the weekly slaughtered reindeer originated from herds where infectious reindeer were actually present during the transmission season, thus allowing condemnation rates to be comparable with the model predicted transmission potential.

We performed a Mann-Kendall (M-K) test [26] to identify trends in the effective transmission period in the time series data (1979–2015) and the potential number of L3 transmitted, then reported the *P*- and tau-values.

Technically, 20% of reindeer could carry the infection from the previous year, as only 80% of animals are dewormed during autumn. We therefore identified the years when liver condemnation was higher than 20% in at least one slaughter batch to assess whether condemnation was due to transmission for that particular year and compared it with the years when the model predicted a higher number of L3 transmitted (≥ 100 worms) (Sensitivity). We also identified the years when our model predicted no/very low worm transmission (< 100) and slaughterhouse data showed a liver condemnation rate under 20% (Specificity). We performed a Chi-square test to identify the diffence between condemnation on different dates or in different cooperatives, and reported the *P*-value.

## Results

We obtained data for 393,161 reindeer slaughtered over 2349 days between 2004 and 2015 at different slaughterhouses in Lapland, with a mean of 169 (range: 10–2720) slaughtered animals each day. Among the slaughtered animals, 82,474 were in the southern region, 135,628 were in the central region and 175,059 were in the northern region. The livers of 11,161 (13.5%) animals were condemned due to *S. tundra* infection in the southern region, 4295 (3.1%) in the central region and 2776 (1.5%) in the northern region.

### The proportion of condemnation in reindeer due to *S. tundra* infection

**Fig. 1:**
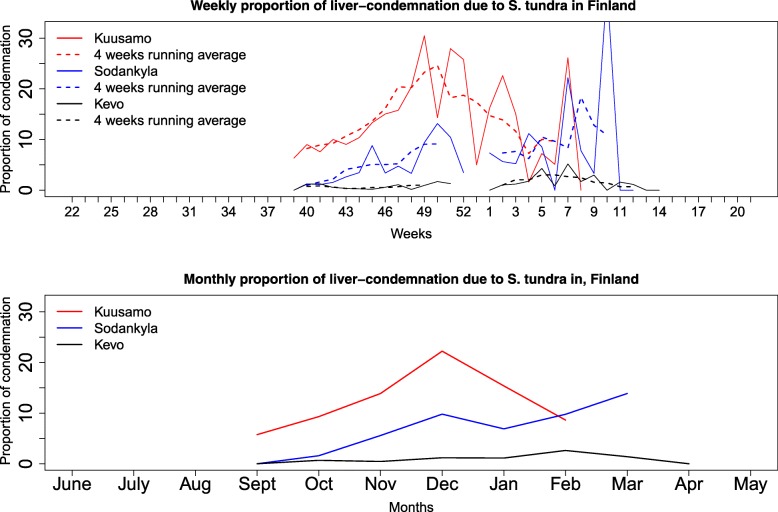
Seasonal variation in the proportion of liver condemnation due to *S. tundra* infection in reindeer. Information was retrieved through slaughterhouse data during meat inspection in different weeks (top panel) and months (bottom panel), 2004–2015. Only a small number of records were available during weeks 1, 52 and 53

The mean proportion of liver condemnation in reindeer across all cooperatives for the period 2004–2015 showed a trend of more frequent condemnation at late slaughter dates (Fig. 1). The proportion of liver condemnation peaked during December in the southern region and March in the central and northern regions (Fig. 1).

**Fig. 2:**
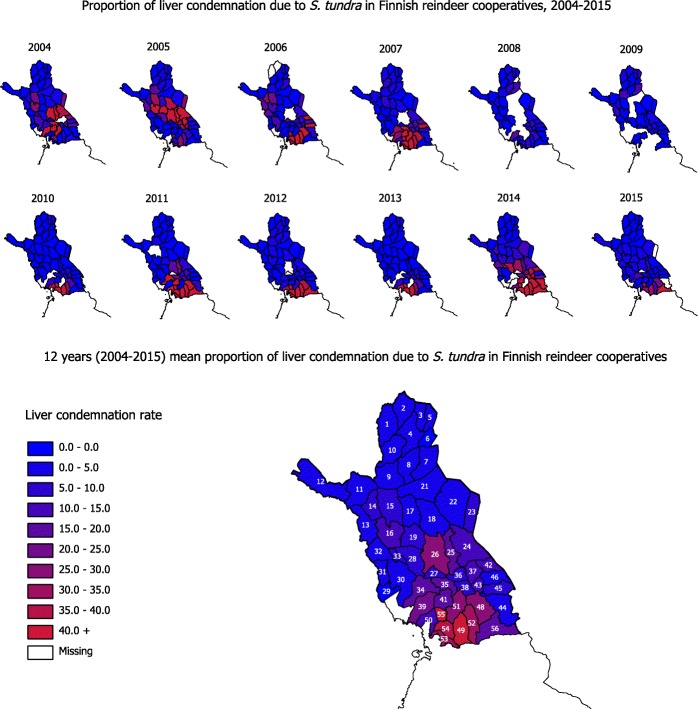
Map of Finnish Lapland showing the proportion of liver condemnation due to *S. tundra* infection based on slaughterhouse inspection data from reindeer cooperatives. The year 2004 here indicates the period between 1st June 2004 and 31st May 2005 and so on. Outbreaks in different cooperatives were grouped together, but there were no fixed foci of outbreaks

Liver condemnation among slaughtered reindeer varied between 0–100% in different years and in different cooperatives (Fig. 2). The mean proportion of liver condemnation in a given year differed between herds sharing the same grazing area, herds being slaughtered on the same day and between the same herds being slaughtered on different days.

We found geographical variation in liver condemnation, with a strong tendency for clustering and a similar proportion of condemned livers in neighboring cooperatives each year. This created hot spots consisting of several cooperatives with spatially clustered outbreaks. Interestingly, the clusters appeared to move around Lapland from year to year. None of the outbreaks affected all of Lapland in any of the investigated years. More outbreaks were recorded in central Lapland (around Sodankylä) in 2004–2007, whereas more outbreaks were recorded in southern Lapland (around Kuusamo) after 2010. Only a small number of outbreaks were recorded in the northern part of Lapland during 2004–2015. Overall, outbreaks were more frequent in the southern part of the reindeer-herding area compared to central or northern Lapland (Fig. 2).

There was a large variation in the daily proportion of liver condemnation among different cooperatives in the same area. Some cooperatives showed high liver condemnation in one batch (animals slaughtered on a specific day), but the following batch (within 4–5 weeks from the first) showed a low proportion of liver condemnation. For example, in the southern region, the proportion of liver condemnation on 18th November 2004 for cooperative number 42 was 61% (*n* = 57; 95% CI: 45.6–77.2%), while 4 weeks later on 15th December, the proportion was significantly lower (*P* < 0.05) at 29% (*n* = 135; 95% CI: 24.7–34.5%). While cooperative 51 had a high proportion of liver condemnation (52–71%) in 2004, a nearby cooperative (48) had no organs condemned in the six slaughter batches in the same year (Additional file 1: Figure S2).

### Model results

#### The proportion of liver condemnation in reindeer *vs* predicted transmitted L3 from an infectious reindeer

**Fig. 3:**
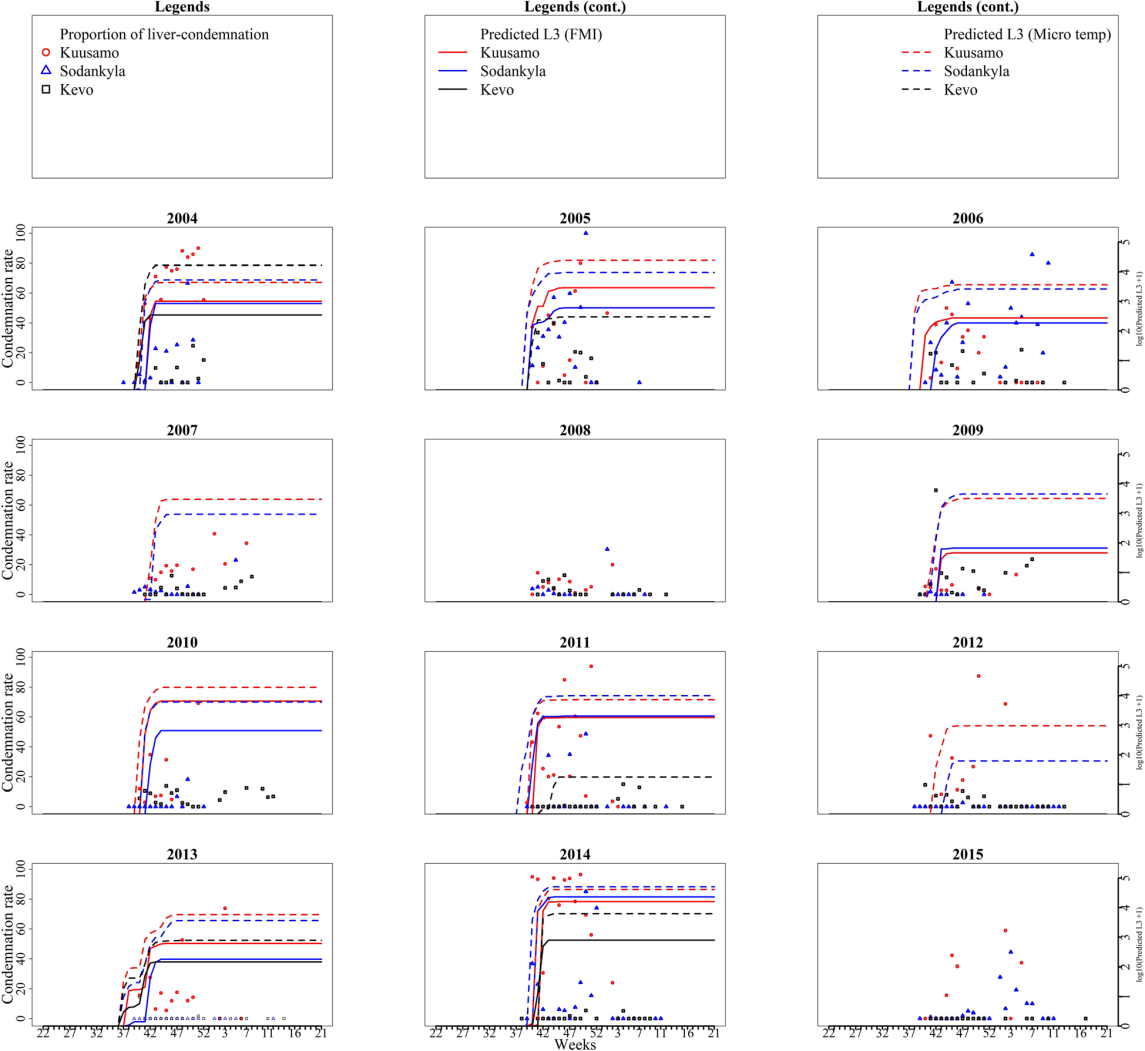
Potential cumulative number of *S. tundra* L3 larvae transmitted from one infectious reindeer and the maximum proportion of liver condemnation due to *S. tundra* in Finnish reindeer per week (based on slaughterhouse observations) in the southern region, Kuusamo (red), the central region, Sodankylä (blue), and the northern region, Kevo (black), of Finnish Lapland, 2004–2015. Note the two Y-axes have different units. The model based on Finnish meteorological temperatures did not detect worm transmission during the years 2007, 2008 and 2015 in Kuusamo (southern region), whereas the model using estimated microclimatic temperatures showed moderate-to-high worm transmission in those years. Liver condemnation rate and predicted number of worm transmitted peaked during the same period.

We used the model to estimate the cumulative number of L3 transmitted per week per infectious reindeer, based on both meteorological and microclimatic temperatures. We also identified the maximum proportion of liver condemnation in each week among the cooperatives in all three regions. A comparison between the predicted cumulative number of L3 transmitted and the maximum proportion of liver condemnation per week in the three different regions is presented in Fig. 3.

A higher proportion of liver condemnation was detected in the southern and central regions than in the northern region for most of the year. Liver condemnation began earlier (September) in the southern region compared to central and northern regions (October). The model predicted that worms mature to cause liver damage at around week 40–42, the same time that liver condemnation also starts to increase in different cooperatives. The models (for both meteorological and microclimatic temperatures) also showed a plateau in the potential number of transmitted mature worms at the same time that the maximum proportion of liver condemnation was reached. The model predicted that worm transmission from vector to host starts and ends within a very short period of time (6–8 weeks) between mid-July and late August. Any vectors biting infectious reindeer outside this period will be infected, but the worms will not be able to mature or be transferred to a new host. The period during which reindeer were infected was shorter than the development time from L3 to new microfilaria, making it impossible for *S. tundra* to have more than one generation each summer. Therefore, all new infections transmitted from mosquitoes to reindeer in one summer originated from reindeer infected the previous summer.

For some years, the model based on meteorological temperature predicted that L3 transmission was not possible, and the model based on microclimatic temperatures predicted it was possible. For example, in 2007 in the southern region, > 40% liver condemnation was observed in slaughtered reindeer, but our FMI-based model suggested that L3 transmission would not be possible. Likewise, the FMI-based model did not predict any potential transmission in 2012, but the microclimatic model predicted moderate L3 transmission. However, the microclimatic temperature-based model predicted that L3 transmission would be likely in some years (e.g. 2009) when liver condemnation was low.

**Fig. 4:**
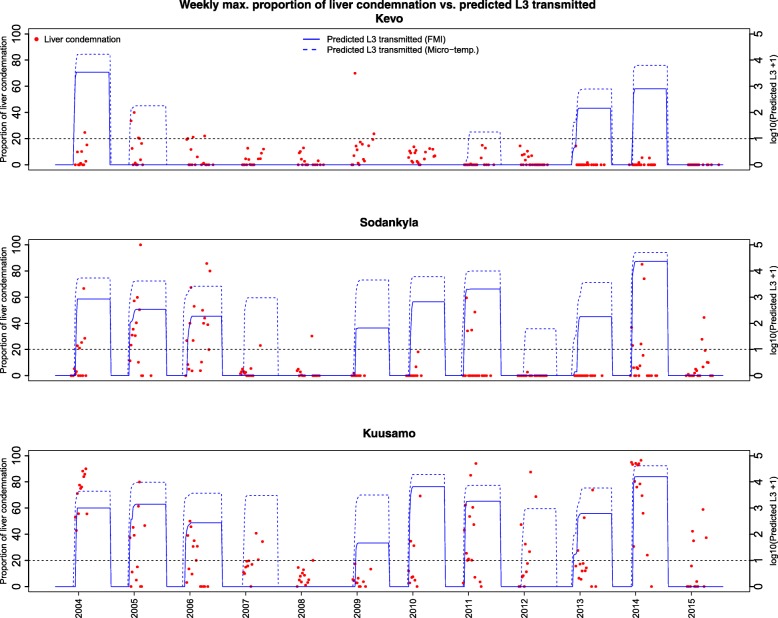
A comparison between the weekly maximum proportion of liver condemnation among the cooperatives in three different regions and the predicted potential transmission of L3 (predicted worm) from an infectious reindeer in the respective cooperatives. The vertical dotted black line (> 20%) indicates the threshold for identifying the year with a higher proportion of worm transmission. Note the two Y-axes have different units. The model driven by Finnish Meteorological Institute temperature showed a higher number of L3 being transmitted in 8 of the 10 years (80%) in the southern region, 6 of the 7 years (86%) in the central region and 1 of the 4 years (25%) in the northern region, coinciding with a high proportion of liver condemnation. The model driven by estimated microclimatic temperature showed a higher number of L3 being transmitted in 9 of the 10 years (90%) in the southern region, 6 of the 7 years (86%) in the central region and 2 of the 4 years (50%) in the northern region, coinciding with a high proportion of liver condemnation

Overall, the model based on FMI temperature predicted a higher number of L3 potentially transmitted in the years when the proportion of liver condemnation was high (Fig. 4). When there was more than 20% liver condemnation in at least one slaughter batch, our model predicted a higher potential L3 transmission in 8 out of 10 years (80%) in the southern region, 6 out of 7 years (86%) in the central region and 1 out of 4 years (25%) in northern region. When less than 20% of livers were condemned, our model showed no/low worm transmission in 1 out of 2 years in the southern region, 3 out of 5 years in the central region and 6 out of 7 years in the northern region (Fig. 4). Across the three regions, our model predicted a higher potential worm transmission in 15 out of 21 occurrences (sensitivity: 71%) when a higher proportion of livers were condemned. Likewise, the model predicted no/very low transmission in 11 of the 16 occurrences (specificity: 73%) when there was no/low liver condemnation.

In all three regions, the model based on estimated microclimatic temperature predicted a higher potential L3 transmission accurately in 18 of 22 occurences (sensitivity: 81%) and predicted no/very low transmission in 8 of the 14 occurrences (specificity: 57%) when there was no/low liver condemnation (Fig. 4).

#### The duration of the effective transmission period from host to vector (model output)

**Fig. 5:**
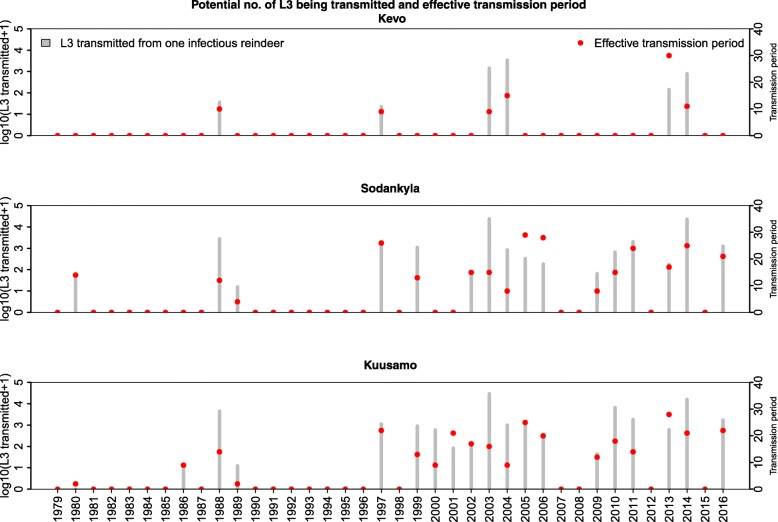
The cumulative number of *S. tundra larvae* L3 transmitted from one infectious reindeer at the end of a season (gray bars). The red dots indicate the duration (in days) of the annual transmission season when mosquitoes receive infected blood meals and survive to successfully transmit the L3 stage of *S. tundra* to a reindeer. Note the two Y-axes have different units

The duration of the effective transmission period and the potential cumulative L3 transmitted from an infectious reindeer are depicted in Fig. 5. The mean duration of the annual effective transmission period was 15.5 days (range: 2–28 days) in the southern region, 17.1 days (range: 4–29 days) in the central region and 14 days (range: 9–30) in the northern region. The M-K test identified a trend of increasing duration over the years (*P*-value < 0.01 for both southern and central regions; tau values of 0.38 for the southern region and 0.36 for the central region). The trend was not significant for the northern region.

The mean (10–90th percentiles) potential number of L3 transmitted each year was on average 1741 (0–2648) in the southern region, 1538 (0–1869) in the central region, and 154 (range: 0–889) in the northern region. The M-K test identified an increasing trend in the potential number of L3 transmitted over the years (*P*-value < 0.01 for the southern region and central region; tau values of 0.36 for the southern region and 0.32 for the central region). The trend was not significant for the northern region.

The longest transmission period was predicted for 2013 in the south, 2005 in the central region and 2014 in the north. The highest number of L3 were transmitted in 2003 in the southern and central regions, and 2004 in the northern region.

## Discussion

There was wide variation in the proportion of liver condemnation between different cooperatives in the same region and within different herds in the same cooperative. There was also wide variation in liver condemnation among the animals slaughtered in the same week within the same cooperatives. The variation may be a result of local differences in mosquito abundance between different herds in the same cooperative, the coverage of anthelmintic administration by the different owners, or the density of reservoir animals (moose, roe deer and forest wild reindeer), but may also be related to differences in observations made by meat inspectors. Because minor changes are not included in the meat inspection decisions, the true incidence of these indicators is likely higher [17]. In other studies, slaughterhouse observations signaled large variation in the proportion of liver condemnation due to cysticercosis in pigs in France [27]. On average, the proportion of liver condemnation due to *S. tundra* infection in reindeer increased gradually over time until December in the southern region and March in the central and northern regions. The reindeer were infected with the L3 stage of *S. tundra* between July and August, and after 2–4 months when these larvae become adults they can cause detectable pathogenesis at slaughter. The reason that the proportion of liver condemnation decreased again after December (in the southern region) or March (in the central and northern regions) is not understood, but may be related to clinical recovery [28].

The estimated L3 transmitted is the potential number of worms originating from one infectious reindeer. When a large number of infectious reindeer are present to infect the vectors, susceptible reindeer will receive more L3 worms and hence be more likely to have organs condemned in the autumn. Therefore, the number of transmitted worms originating from one reindeer as predicted by the model is not directly comparable to the proportion of liver condemnation, which instead reflects the accumulated number of worms received by one reindeer. The actual abundance of vectors was not available for the particular years in this study, but is likely to vary. Our model assumed infectious reindeer to have a specific daily density of microfilariae that would be the same for all years and regions, which may not be the case.

We compared the two independent datasets of the predicted accumulated L3 transmitted per infectious reindeer and the observed condemnation. We expected them to correlate, though not perfectly, because: (i) the data were not equivalent; (ii) the data could not be expected to be linearly correlated (proportion of liver condemnation is 0–100% and potential L3 transmission is unlimited); (iii) we do not know how many worms must be present before condemnation will occur; (iv) there is a potential human bias when identifying the pathology caused by *S. tundra* in different slaughterhouses; and (v) we do not know how many reindeer are actually infected at the start of the season, as this depends on last year’s transmission, the deworming treatment and slaughter rates during winter. Despite these limitations, the model could predict the occurrence of outbreaks fairly well (sensitivity: 71–81%; specificity: 57–73%).

Our model suggested higher transmission rates in the southern region, with transmission starting earlier and lasting longer than in the northern region. This correlated very well with the proportion of liver condemnation. Furthermore, the model detected a positive trend in the duration of the annual transmission period and potential number of worm transmitted in the southern and central regions, which corresponded very well to slaughterhouse findings. Overall, our predictions of potential worm transmission each season matched fairly well with the observed weekly maximum proportion of liver condemnation in reindeer (except in the northern region). However, our model based on FMI temperature did not predict outbreaks in some of the years when outbreaks occurred. For example, in 2007, our model showed transmission would not be possible in the southern region, but inspectors at the slaughterhouses detected a large outbreak. However, when using microclimatic temperature to drive the model, a high L3 transmission was predicted for that year. These findings indicate that microclimatic temperature may play an important role in the local transmission of *S. tundra* in reindeer, as has been found for other vector-borne infections [10, 15, 29].

Our model showed that there is only one transmission cycle possible in any year within the reindeer-herding areas in Lapland, as the L3 larval stage requires a long time (90–120 days) to develop into adult worms in the reindeer and produce microfilaria. Transmission is therefore entirely dependent on some reindeer with microfilaria escaping the deworming treatment during the winter period. Therefore, the model suggests it is possible to prevent outbreaks by deworming all infected reindeer during winter. This is an important finding that could facilitate decision making in terms of the prevention and control of *S. tundra* in Finnish reindeer. Around 80% (range: 64–90%) of reindeer are currently dewormed with the anthelmintic drug ivermectin during the autumn round-up (September to November) [28]. This allows for at least 20% of the animals to act as carriers of the parasite. For *S. tundra* infection in reindeer, there is a linear relationship between the proportion of animals that escape deworming during winter and the number of L3 worms transmitted to susceptible animals the following summer.

Our model identified an increasing trend for both the duration of the effective transmission season as well as the potential worm transmission for the period 1979–2015 for the southern and central regions. This finding agrees with the proportion of liver condemnation observed during 2004–2015, when there were a number of large outbreaks recorded in these areas. However, only a small number of outbreaks were identified in the northern region during this period, and our model showed a non-significant trend in the northernmost regions of Lapland. Approximately 30–40% of southern and central regions have wetland and bogs rich in water suitable for mosquito breeding, whereas bog habitats make up only around 12% of the northern region [30]. Due to the geographical location, this northern region is generally much colder than the other studied areas (Additional file 1: Figure S3). Furthermore, in northern areas there are more open and windy spaces, and snowy mountains, which is expected to allow reindeer to escape vectors, thus potentially resulting in lower biting rates [31]. This could result in lower vector abundance and thus cause only small outbreaks in the northern region, while the model calculations assumed identical vector densities across all three regions and all years.

There was a low proportion of liver condemnation in 2008 in the southern region. Our FMI-based model predicted no transmission being possible in 2008, as the temperature was not high enough. In a situation like this, the abundance of mosquitoes will have no impact on model output. Our model also showed that an outbreak is only possible if the mosquitoes are infected early in the season (early May to mid-July) (Additional file 1: Figure S4), so they can survive long enough to complete the EIP. However, the extent of the outbreak will depend on the timing of the peak period of mosquito abundance, microfilarial density and temperature. Warm summers alone therefore may not be enough for a large *S. tundra* outbreak in Finland, as the timing of warm periods, high mosquito abundance and microfilarial density also plays a role.

Our model predicted higher worm transmission in 71–81% of cases with a high proportion of organ condemnation, and lower or no transmission in 57–73% of cases with low/no liver condemnation. This means that our model correlated with the true outbreaks very well, but did not correlate well for years with no outbreaks. A possible reason for this could be that *S. tundra* in Finland can be transmitted at lower temperatures than other filaraoid nematodes (14 °C) [12]. Our model could not capture this because the equation we used was not specific to *S. tundra* and the temperature-dependent development rate of *S. tundra* might be different from other filarioid nematodes. Another possible reason could be that microclimatic temperatures are higher than we estimated.

There are no published data on mosquito life history (biting rate, survival rate, life span, etc.) available from Finland, and the formulae we used for the biting rate of mosquitoes were based on studies using laboratory conditions in tropical countries (Thailand and Puerto Rico). The mathematical calculation of EIP used in this study was originally developed from *Dirofilaria immitis*. We developed an independent biological process-based model using parameter estimates from the literature, rather than a statistical model fitted to the outbreak data [3]. The model is therefore independent of the condemnation data, and it is interesting that the predicted L3 transmission from one infectious reindeer correlates well with the observed liver condemnation data. We assumed that all microfilariae ingested by a mosquito would become L3 if the mosquito survived long enough, and that all L3 larvae would be transmitted to a new host. In reality, only a fraction of the microfilariae in a mosquito will become L3, and only a small fraction of these will be transmitted to a new host.

## Conclusions

There was a geographical variation in the proportion of liver condemnation due to *S. tundra* infection across different reindeer-herding areas and in different years with more outbreaks being recorded in southern region. Temperature was an important driver of *S. tundra* outbreaks in Finnish reindeer. We found the effective transmission period for *S. tundra* to be very short for reindeer in Lapland, but it showed an increasing trend over the period. Only one generation of transmission was possible in each season, indicating the possibility of controlling *S. tundra* outbreaks in Finland by deworming all animals effectively at the end of summer. The model predicted higher worm transmission when a larger proportion of organs were condemned (sensitivity: 71–81 %) due to *S. tundra* infection, and lower or no transmission when low/no liver condemnation was recorded (specificity: 57–73%).

## Additional files

Additional file 1: **Figure S1.** The daily abundance of mosquitoes [1] and density of microfilaria per ml of blood from reindeer in Oulu Zoo [2] in 2004, used for the *S. tundra* transmission model. **Figure S2.** The proportion of liver condemnation across different cooperatives in the southern region, Kuusamo (circles), central region, Sodankylä (triangles) and northern region, Kevo (rectangles) from 2004 to 2015. **Figure S3.** The annual mean temperature for the summer period (June-August) in the southern (Kuusamo), central (Sondakyla), and northern (Kevo) regions of Lapland. **Figure S4.** The estimated period when *S. tundra* microfilariae can be transmitted from reindeer to mosquito vectors. Only microfilariae that were successful in becoming L3 *S. tundra* from the same vector are shown. The estimated microfilariae transmitted from one infectious reindeer at three locations in Lapland, i.e. northern (Kevo), central (Sodankylä) and southern (Kuusamo), are presented by the date when the mosquitoes received the microfilaria-infected blood meal. The dates the reindeer are infected with L3 cannot be seen from this graph. Central and southern regions had the longest duration of transmission period and the largest number of microfilariae transmitted in 2014 (cyan, dotted), whereas transmission for the northern region (Kevo) peaked in 2004 (blue). (DOCX 568 kb)

## Abbreviations

EIP: Extrinsic incubation period
FMI: Finish Meteorological Institution
M-K test: Mann-Kendall test
