# Supplementary material for: The annual, temporal and spatial pattern of Setaria tundra outbreaks in Finnish reindeer: a mechanistic transmission model approach
Source: Parasit Vectors. 2018 Nov 12;11:565. doi: 10.1186/s13071-018-3159-z (PMC6231263; doi:10.1186/s13071-018-3159-z)
Supplement: Supplementary file 1 — Figure S1. The daily abundance of mosquitoes [1] and density of microfilaria per ml of blood from reindeer in Oulu Zoo [2] in 2004, used for the S. tundra transmission model. Figure S2. The proportion of liver condemnation across different cooperatives in the southern region, Kuusamo (circles), central region, Sodankylä (triangles) and northern region, Kevo (rectangles) from 2004 to 2015. Figure S3. The annual mean temperature for the summer period (June-August) in the southern (Kuusamo), central (Sondakyla), and northern (Kevo) regions of Lapland. Figure S4. The estimated period when S. tundra microfilariae can be transmitted from reindeer to mosquito vectors. Only microfilariae that were successful in becoming L3 S. tundra from the same vector are shown. The estimated microfilariae transmitted from one infectious reindeer at three locations in Lapland, i.e. northern (Kevo), central (Sodankylä) and southern (Kuusamo), are presented by the date when the mosquitoes received the microfilaria-infected blood meal. The dates the reindeer are infected with L3 cannot be seen from this graph. Central and southern regions had the longest duration of transmission period and the largest number of microfilariae transmitted in 2014 (cyan, dotted), whereas transmission for the northern region (Kevo) peaked in 2004 (blue). (DOCX 568 kb) [file 13071_2018_3159_MOESM1_ESM.docx]

**The annual, temporal and spatial pattern of *Setaria tundra* outbreaks in Finnish reindeer: a mechanistic transmission model approach**

Najmul Haider^1*^, Sauli Laaksonen^2,3^, Lene Jung Kjær^1^, Antti Oksanen^4^ and Rene Bødker^1^

^1^National Veterinary Institute, Technical University of Denmark, Kgs. Lyngby, Denmark.

^2^Department of Veterinary Biosciences, Faculty of Veterinary Medicine, University of Helsinki, Helsinki, Finland.

^3^WAZAMA Media Oy, Kuusamo, Finland.

^4^Finnish Food Safety Authority Evira (FINPAR), Oulu, Finland.

*Correspondence: [najha@vet.dtu.dk](mailto:najha@vet.dtu.dk)

**Additional file 1: Figures S1-S4**

**Additional file 1: Figure S1. The daily abundance of mosquitoes** [1], **and density of microfilaria per ml of blood from reindeer in Oulu Zoo** [2] **in 2004, used for the *S. tundra* transmission model.**

**Additional file 1: Figure S2. The proportion of liver condemnation across different cooperatives in the southern region, Kuusamo (circles), central region, Sodankylä (triangles) and northern region, Kevo (rectangles) from 2004-2015.**

| **** |
| --- |
|  |

**Additional file 1: Figure S3. The annual mean temperature for the summer period (June-August) in the southern (Kuusamo), central (Sondakyla), and northern (Kevo) regions of Lapland.**

**Additional file 1: Figure S4. The estimated period when *S. tundra* microfilaria can be transmitted from reindeer to mosquito vectors. Only microfilaria that were successful in becoming L3 *S. tundra* from the same vector is shown. The estimated microfilaria transmitted from one infectious reindeer at three locations in Lapland: northern (Kevo), central (Sodankylä) and southern (Kuusamo) are presented by the date when the mosquitoes received the microfilaria infected blood meal. The dates the reindeer are infected with L3 cannot be seen from this graph. Central and southern regions had the longest duration of transmission period and the largest number of microfilaria transmitted in 2014 (cyan, dotted), whereas transmission for the northern region (Kevo) peaked in 2004 (blue).**

References:

1. Laaksonen S. *Setaria tundra*, an emerging parasite of reindeer, and an outbreak it caused in Finland in 2003–2006, PhD thesis. University of Helsinki, Finland; 2010.
2. Laaksonen S, Solismaa M, Orro T, Kuusela J, Saari S, Kortet R, et al. *Setaria tundra* microfilariae in reindeer and other cervids in Finland. Parasitol Res. 2009;104:257–65.
